# Prediction of risk factors for respiratory failure in children with mycoplasma pneumoniae pneumonia based on clinical pulmonary infection scores

**DOI:** 10.3389/fped.2026.1891146

**Published:** 2026-08-28

**Authors:** Jiao Lian, Xiyao Si, Xiaolong Li, Tao Xue, Zhenying Liu

**Affiliations:** Pediatric Internal Medicine, Yulin City Children’s Hospital, Yuyang District People’s Hospital of Yulin City, Yulin, Shaanxi, China

**Keywords:** clinical pulmonary infection score, mycoplasma pneumoniae pneumonia, nomogram, respiratory failure, risk prediction

## Abstract

**Background:**

Mycoplasma pneumoniae pneumonia (MPP) remains a leading cause of community-acquired pneumonia in children, with respiratory failure occurring in 15%–20% of hospitalized cases. Despite advances in understanding MPP pathogenesis, validated tools for early prediction of respiratory failure are lacking. This study aimed to develop and validate a nomogram based on Clinical Pulmonary Infection Score (CPIS) for individualized risk stratification in pediatric MPP.

**Methods:**

We conducted a retrospective cohort study of 305 children with confirmed MPP admitted to our hospital between July 2020 and December 2024. Patients were categorized into respiratory failure (*n* = 62) and non-respiratory failure (*n* = 243) groups. Demographics, clinical features, laboratory parameters, chest imaging findings, and CPIS were collected. Independent risk factors were identified through multivariate logistic regression, and a nomogram was constructed and validated using ROC curve analysis, calibration plots, and decision curve analysis.

**Results:**

Respiratory failure was associated with significantly higher rates of tachypnea (91.9% vs. 16.5%), interstitial changes (87.1% vs. 25.5%), and pleural effusion (74.2% vs. 24.7%) (all *P* < 0.001). The respiratory failure group exhibited elevated NLR (5.2 vs. 3.7), CRP, D-dimer, and CPIS (7.4 vs. 5.3), with lower SpO_2_ (89.6% vs. 94.3%) (all *P* < 0.001). Multivariate logistic analysis identified interstitial changes (OR = 2.522, *P* = 0.001), NLR (OR = 2.716, *P* < 0.001), and CPIS (OR = 1.932, *P* < 0.001) as independent risk factors, while older age (OR = 0.467, *P* < 0.001) and higher SpO_2_ (OR = 0.772, *P* < 0.001) were protective. The nomogram demonstrated exceptional discriminative ability (AUC = 0.918, 95% CI: 0.880–0.956), good calibration (Hosmer-Lemeshow *P* = 0.71), and positive net benefit across threshold probabilities of 5%–40%.

**Conclusions:**

This CPIS-based nomogram provides a highly accurate, clinically practical tool for predicting respiratory failure in pediatric MPP. By integrating radiographic, inflammatory, and physiological parameters, it enables early identification of high-risk patients for enhanced monitoring and preemptive respiratory support, potentially improving outcomes in severe MPP.

## Introduction

Mycoplasma pneumoniae pneumonia (MPP) is a major cause of community-acquired pneumonia in children, accounting for 10%–40% of pediatric cases and showing epidemic cycles every 3–7 years (1). As an important pathogen of community-acquired pneumonia (CAP) in the pediatric population, Mycoplasma pneumoniae (MP) can lead to a wide spectrum of clinical manifestations, ranging from mild upper respiratory tract infections to severe pneumonia and even respiratory failure (2). Although MPP typically presents with mild and self-limiting respiratory symptoms, a considerable proportion of pediatric patients develop severe disease, characterized by refractory hypoxemia, acute respiratory distress syndrome (ARDS), and respiratory failure, which may necessitate intensive care unit (ICU) admission and mechanical ventilation (3). Moreover, recent epidemics driven by macrolide-resistant MPP strains have increased the incidence of severe complications, with respiratory failure occurring in 15%–20% of hospitalized children (4). Such disease progression not only raises mortality risk but also imposes a significant burden on healthcare resources, highlighting the crucial need for early risk stratification.

However, translating this need into clinical practice remains challenging. Despite advances in understanding MPP pathogenesis, clinical practice still lacks validated, user-friendly tools to predict respiratory failure at the time of hospital admission (5). Current risk assessment relies predominantly on physician experience and isolated biomarkers such as C-reactive protein (CRP) or lactate dehydrogenase (LDH), which demonstrate modest predictive accuracy (AUC: 0.70–0.80) and fail to capture the multifactorial nature of disease severity (6). The Clinical Pulmonary Infection Score (CPIS), originally developed for ventilator-associated pneumonia, integrates clinical, radiographic, and physiological parameters into a composite severity index (7). Although CPIS has shown promise in adult pneumonia cohorts, its application and predictive value in pediatric MPP remain largely unexplored. Similarly, the neutrophil-to-lymphocyte ratio (NLR) has emerged as a robust, cost-effective marker of systemic inflammation and immune dysregulation in various infectious diseases (8), yet its independent contribution to MPP severity prediction remains undefined. However, subjective interpretation and lack of standardized integration with clinical parameters limit its standalone utility for risk prediction. We hypothesized that a multivariable model combining CPIS with objective inflammatory (NLR) and radiographic markers would substantially improve prediction of respiratory failure in pediatric MPP. Therefore, this study aimed to develop and validate a nomogram-based prediction model to enable early identification of high-risk children, facilitate timely escalation of care, and optimize resource allocation through personalized risk stratification.

## Materials and methods

### Study design and participants

We conducted a retrospective cohort study of children admitted with Mycoplasma pneumoniae pneumonia (MPP) to Yulin City Children's Hospital, a tertiary pediatric referral center, between July 2020 and December 2024. All human samples were obtained with informed consent in accordance with the Declaration of Helsinki.

Inclusion criteria: Patients aged 1–14 years. Patients with at least one of the following clinical pneumonia symptoms: (1) positive Mycoplasma pneumoniae PCR from nasopharyngeal aspirate or throat swab, (2) seroconversion with a four-fold rise in IgG antibody titer, or (3) single IgM antibody titer >1:160. Respiratory failure was defined as the need for invasive or non-invasive mechanical ventilation within 72 h of admission, or a partial pressure of oxygen to fraction of inspired oxygen (PaO_2_/FiO_2_) ratio <300 mmHg despite supplemental oxygen. Patients performed chest radiographs within 48 h of admission.

Exclusion criteria: (1) known immunodeficiency disorders, (2) concurrent bacterial or viral co-infections confirmed by multiplex PCR, (3) chronic lung diseases (e.g., bronchopulmonary dysplasia, cystic fibrosis), (4) congenital heart disease, (5) incomplete medical records or missing critical laboratory data, and (6) transfer from other hospitals with >24 h of prior treatment.

### Data collection

Demographic and clinical data were extracted from electronic medical records using a standardized case report form. Collected variables included age, sex, presence of high fever (≥38.5 ℃), cough, tachypnea (respiratory rate >50 breaths/min for age <6 years, >30 breaths/min for age ≥6 years), and vital signs at admission.

Laboratory parameters obtained within 24 h of admission included: white blood cell count (×10^9^/L), platelet count (×10^9^/L), hemoglobin (g/L), neutrophil-to-lymphocyte ratio (NLR), C-reactive protein (CRP, mg/L), procalcitonin (PCT, ng/mL), lactate dehydrogenase (LDH, U/L), albumin (g/L), fibrinogen degradation products (FDP, mg/L), and D-dimer (mg/L). Oxygen saturation (SpO_2_) was recorded by pulse oximetry on room air or at documented FiO_2_.

Chest radiographs performed within 48 h of admission were independently reviewed by two pediatric radiologists blinded to clinical outcomes. Discrepancies were resolved by consensus. Imaging findings were categorized as presence or absence of: (1) interstitial changes (reticular opacities, septal thickening), (2) pulmonary consolidation (air bronchograms, lobar consolidation), (3) pleural effusion, and (4) other pulmonary opacities.

The Clinical Pulmonary Infection Score (CPIS) was calculated at admission based on six components: fever, leukocytosis, tracheal secretions, oxygenation (PaO_2_/FiO_2_), radiographic infiltrates, and culture results, with scores ranging from 0 to 12 as previously described (9). Given that tracheal secretion assessment is impractical in non-intubated children, we utilized a modified CPIS excluding the tracheal secretion component. The modified CPIS comprised four components: (1) temperature, (2) peripheral white blood cell count, (3) oxygenation (PaO_2_/FiO_2_), and (4) chest radiographic infiltrates, with total scores ranging from 0 to 10.

### Statistical analysis

Statistical analyses were performed using SPSS version 26.0 (IBM Corp., Armonk, NY) and R software (R Foundation for Statistical Computing, Vienna, Austria). Categorical variables were presented as frequencies and percentages, analyzed by *χ*^2^ or Fisher's exact tests. Continuous variables were expressed as mean ± standard deviation (SD) or median with interquartile range (IQR) based on distribution normality, and compared using independent t-tests or Mann–Whitney U tests. Correlation analysis between CPIS and continuous variables was performed using Pearson correlation coefficients.

Univariate and multivariate logistic regression was conducted to screen potential predictors. Model performance was assessed by the area under the ROC curve (AUC) with 95% confidence intervals (CI). Internal validation was performed using 1,000 bootstrap resamples to correct for overfitting. To address potential physiological overlap between the outcome definition (which includes PaO_2_/FiO_2_ < 300 mmHg) and oxygenation-related predictors (SpO_2_ and the oxygenation component of the modified CPIS), a sensitivity analysis was conducted. In this sensitivity model, SpO_2_ and the PaO_2_/FiO_2_ item were removed from the modified CPIS (recalculating the score on a 0–8 point scale).

A nomogram was constructed based on the multivariate regression coefficients using the rms package in R software version 8.1. Calibration curves were generated to evaluate agreement between predicted probabilities and observed outcomes, with the Hosmer-Lemeshow test applied (*P* > 0.05 indicating good calibration). Decision curve analysis quantified the clinical utility by calculating net benefit across various risk thresholds. All statistical comparisons were two-sided, with *P* < 0.05 considered significant.

## Results

### Comparison of basic characteristics between the respiratory failure and non-respiratory failure groups

A total of 305 patients with mycoplasma pneumoniae pneumonia (MPP) were enrolled, including 243 in the non-respiratory failure group and 62 in the respiratory failure group. The baseline demographic and clinical characteristics are summarized in Table 1. The mean age was comparable between the non-respiratory failure and respiratory failure groups (*P* < 0.001). No significant difference was observed in sex distribution, with males accounting for 52.7% (128/243) and 53.2% (33/62) of the two groups, respectively (*P* = 0.938).

**Table 1:** Comparison of basic characteristics between the respiratory failure and non-respiratory failure.

Indicator	Non-respiratory failure (243)	Respiratory failure (62)	*P*
Age	5.6 (3.3, 8.5)	2.0 (0.6, 3.0)	<0.001
Gender			
Male	128	33	0.938
Female	115	29	
Hyperpyrexia			
No	15	7	0.164
Yes	228	55	
Cough			
No	3	1	0.815
Yes	240	61	
Tachypnea			
No	203	5	<0.001
Yes	40	57	
Interstitial changes			
No	181	8	<0.001
Yes	62	54	
Pulmonary consolidation			
No	101	6	<0.001
Yes	142	56	
Pleural effusion			
No	220	21	<0.001
Yes	23	41	
SpO2	94.3 ± 3.6	89.6 ± 5.9	<0.001
CPIS	5.3 ± 1.9	7.4 ± 1.9	<0.001

Regarding clinical symptoms, the prevalence of high fever did not differ significantly between groups [93.8% [228/243] vs. 88.7% [55/62], *P* = 0.164]. Similarly, the proportion of patients with cough was comparable [98.8% [240/243] vs. 98.4% [61/62], *P* = 0.815]. However, the incidence of tachypnea was significantly higher in the respiratory failure group [91.9% [57/62] vs. 16.5% [40/243], *P* < 0.001].

Chest imaging findings revealed significant inter-group differences. Interstitial changes were present in 87.1% (54/62) of patients with respiratory failure vs. 25.5% (62/243) in those without (*P* < 0.001). Pulmonary consolidation was also more frequent in the respiratory failure group [90.3% [56/62] vs. 58.4% [142/243], *P* < 0.001]. Additionally, pleural effusion occurred more commonly among patients who developed respiratory failure [74.2% [46/62] vs. 24.7% [60/243], *P* < 0.001]. Oxygen saturation (SpO_2_) was markedly reduced among patients with respiratory failure (89.6 vs. 94.3, *P* < 0.001), while the Clinical Pulmonary Infection Score (CPIS) was significantly elevated (7.4 vs. 5.3, *P* < 0.001).

### Comparison of blood indicators between the respiratory failure and non-respiratory failure groups

Hematological indices revealed that respiratory failure group exhibited significantly elevated white blood cell counts (8.1 vs. 7.2, *P* = 0.026), neutrophil-to-lymphocyte ratio (NLR, 5.2 vs. 3.7, *P* < 0.001), C-reactive protein levels (17.8 vs. 15.8, *P* = 0.015), and D-dimer concentrations (2.6 vs. 1.9, *P* < 0.001) compared to the non-respiratory failure group. Hemoglobin concentrations were significantly lower in the respiratory failure group (11.7 vs. 15.1, *P* = 0.027). Platelet counts were 333.1 in the respiratory failure group and 274.9 in the non-respiratory failure group; however, statistical significance was not reported. No significant differences were observed for fibrinogen degradation products (2.2 vs. 1.8, *P* = 0.239), procalcitonin (2.2 vs. 1.9, *P* = 0.433), lactate dehydrogenase (397.4 vs. 243.5, *P* = 0.072), or albumin (41.2 vs. 43.4, *P* = 0.086) (Table 2).

**Table 2:** Comparison of blood indicators between the respiratory failure and non-respiratory failure.

Indicator	Non-respiratory failure (243)	Respiratory failure (62)	*P*
WBC	7.2 ± 1.6	8.1 ± 1.7	0.026
PLT	274.9 ± 102.4	333.1 ± 97.7	0.137
NLR	3.7 ± 1.1	5.2 ± 1.3	<0.001
D-D	1.9 ± 0.8	2.6 ± 1.3	<0.001
FDP	1.8 ± 1.0	2.2 ± 0.9	0.239
CRP	15.8 ± 3.7	17.8 ± 2.6	0.015
PCT	1.9 ± 0.4	2.2 ± 0.7	0.433
LDH	243.5 ± 95.6	397.4 ± 73.4	0.072
Hb	15.1 ± 4.0	11.7 ± 1.9	0.027
Alb	43.4 ± 2.8	41.2 ± 2.7	0.086

WBC, white blood cell count; PLT, blood platelet count; NLR, neutrophil to lymphocyte ratio; D-D, D-dimer; FDP, fibrinogen degradation products; CRP, C-reactive protein; PCT, procalcitonin; LDH, lactate dehydrogenase; Hb, hemoglobin; Alb, albumin.

### Correlation between CPIS and respiratory failure-related indicators

**Figure 1:**
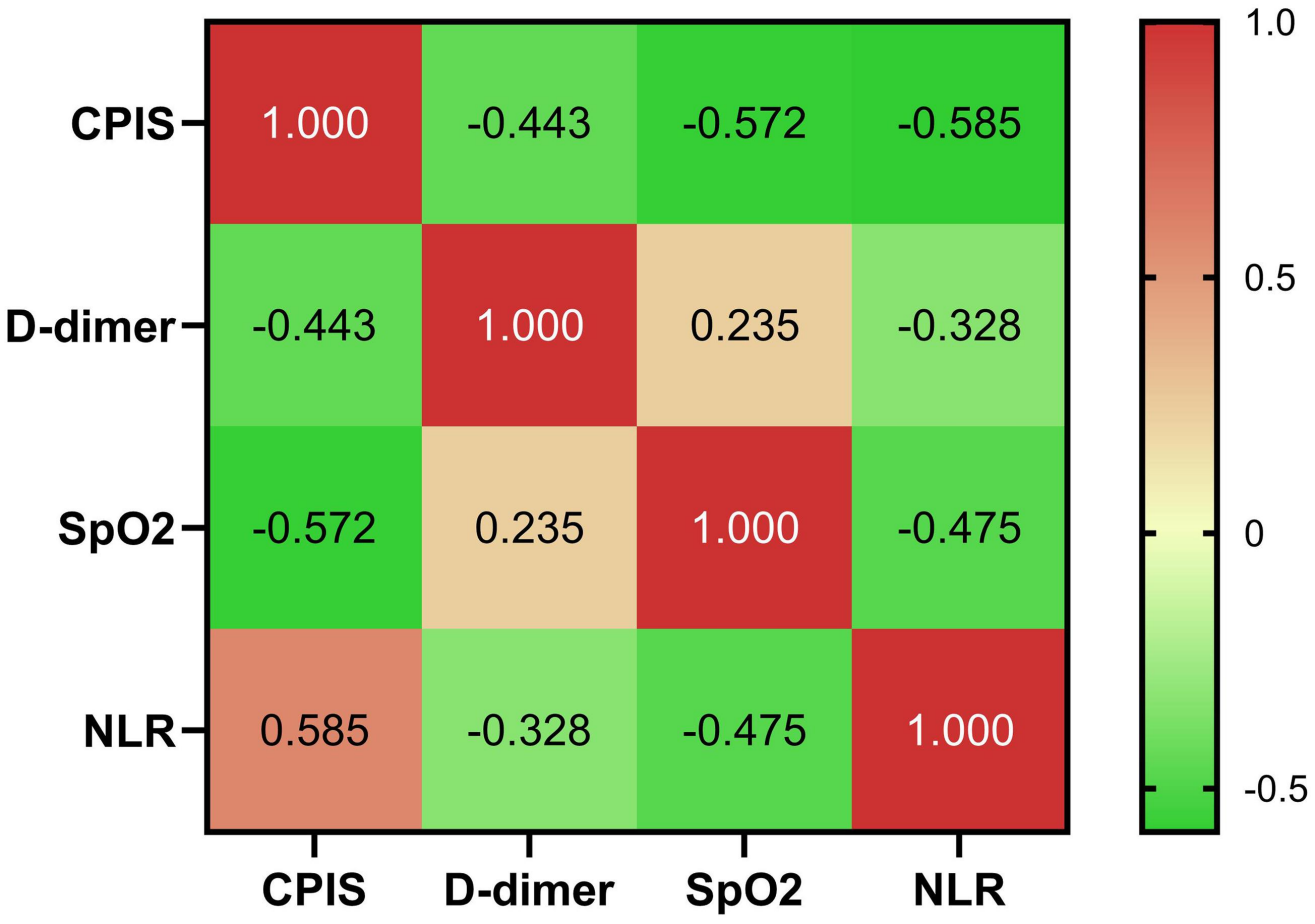
Correlation between CPIS and respiratory failure related indicators.

The correlation analysis results are shown in Figure 1. CPIS is significantly negatively correlated with SpO_2_ (*r* = −0.572), significantly positively correlated with NLR (*r* = 0.585), and moderately negatively correlated with D-dimer (*r* = −0.443). In addition, there is a negative correlation between SpO_2_ and NLR (*r* = −0.475). All correlation coefficients have statistical significance (*P* < 0.05).

### Risk factors for respiratory failure

Univariate and multivariate logistic regression analyses were performed to identify independent predictors of respiratory failure (Table 3). In univariate analysis, multiple variables demonstrated significant associations, including age, tachypnea, radiographic features (pulmonary opacities, interstitial changes, consolidation, pleural effusion), leukocyte count, platelet count, NLR, D-dimer, FDP, CRP, PCT, LDH, hemoglobin, albumin, SpO_2_, and CPIS (all *P* < 0.05).

**Table 3:** Risk factors for respiratory failure.

	Univariate analysis					Multivariate analysis				
Indicator	B	*P*	OR	95% C.I. of OR		B	*P*	OR	95% C.I. of OR	
				Lower limit	Upper limit				Lower limit	Upper limit
Age	−0.656	<0.001	0.519	0.430	0.627	−0.762	<0.001	0.467	0.337	0.646
Gender	0.022	0.938	1.022	0.585	1.788					
Hyperpyrexia	−0.660	0.171	0.517	0.201	1.329					
Cough	−0.271	0.816	0.763	0.078	7.459					
Tachypnea	4.058	<0.001	57.855	21.822	153.385					
Interstitial changes	2.981	<0.001	19.706	8.885	43.705	1.709	0.001	2.522	1.954	15.610
Pulmonary consolidation	2.171	<0.001	8.769	4.627	16.617					
Pleural effusion	2.927	<0.001	18.675	9.470	36.827					
WBC	0.349	<0.001	1.418	1.192	1.687					
PLT	0.006	<0.001	1.006	1.003	1.009					
NLR	1.130	<0.001	3.014	2.207	4.115	0.999	<0.001	2.716	1.685	4.378
D-dimer	0.862	<0.001	2.368	1.705	3.289					
FDP	0.383	0.005	1.467	1.122	1.918					
CRP	0.173	<0.001	1.189	1.085	1.302					
PCT	0.882	0.003	2.416	1.352	4.316					
LDH	0.007	<0.001	1.007	1.003	1.010					
Hb	−0.298	<0.001	0.742	0.669	0.823					
Alb	−0.208	<0.001	0.813	0.733	0.901					
SpO2	−0.224	<0.001	0.800	0.744	0.760	−0.259	<0.001	0.772	0.682	0.873
CPIS	0.565	<0.001	1.760	1.477	2.096	0.658	<0.001	1.932	1.405	2.655

Multivariate logistic regression analysis revealed that interstitial changes on chest imaging (OR = 2.522, *P* = 0.001), NLR (OR = 2.716, *P* < 0.001), and CPIS (OR = 1.932, *P* < 0.001) were independent risk factors for respiratory failure. Conversely, age (OR = 0.467, *P* < 0.001) and SpO_2_ (OR = 0.772, *P* < 0.001) were independently associated with a reduced risk of respiratory failure. To assess potential multicollinearity among the included variables, we calculated the variance inflation factor (VIF) for each predictor in the multivariate model. The VIF values all well below the conventional threshold of 5, indicating no significant multicollinearity among the final model variables.

### Nomogram for predicting respiratory failure risk model

**Figure 2:**
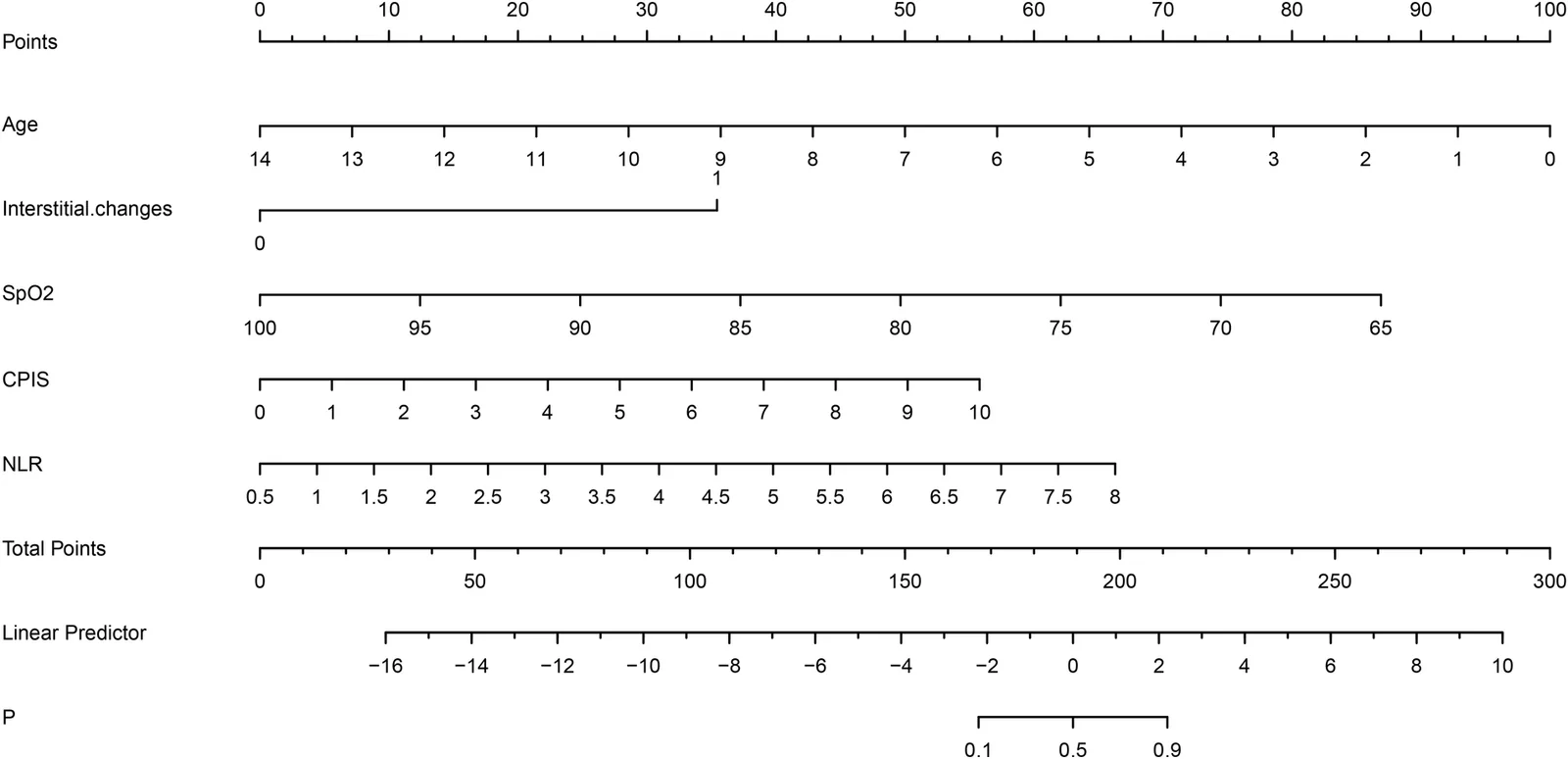
Nomogram for predicting respiratory failure risk model.

Based on the independent predictors identified by multivariate logistic regression analysis, a nomogram was developed to provide a quantitative tool for individualized risk assessment of respiratory failure (Figure 2). The nomogram integrated five key variables: age, SpO_2_, CPIS, NLR, and interstitial changes on chest imaging.

In this nomogram, each variable is assigned a point value proportionate to its regression coefficient. The points for each variable are summed to generate a total score (range: 0–300), which is then projected vertically to the “Linear Predictor” axis and subsequently to the probability of respiratory failure. This visualization tool enables clinicians to quantitatively estimate individualized risk and facilitate early identification of high-risk patients for targeted intervention.

### Clinical predictive efficacy of the respiratory failure model

**Figure 3:**
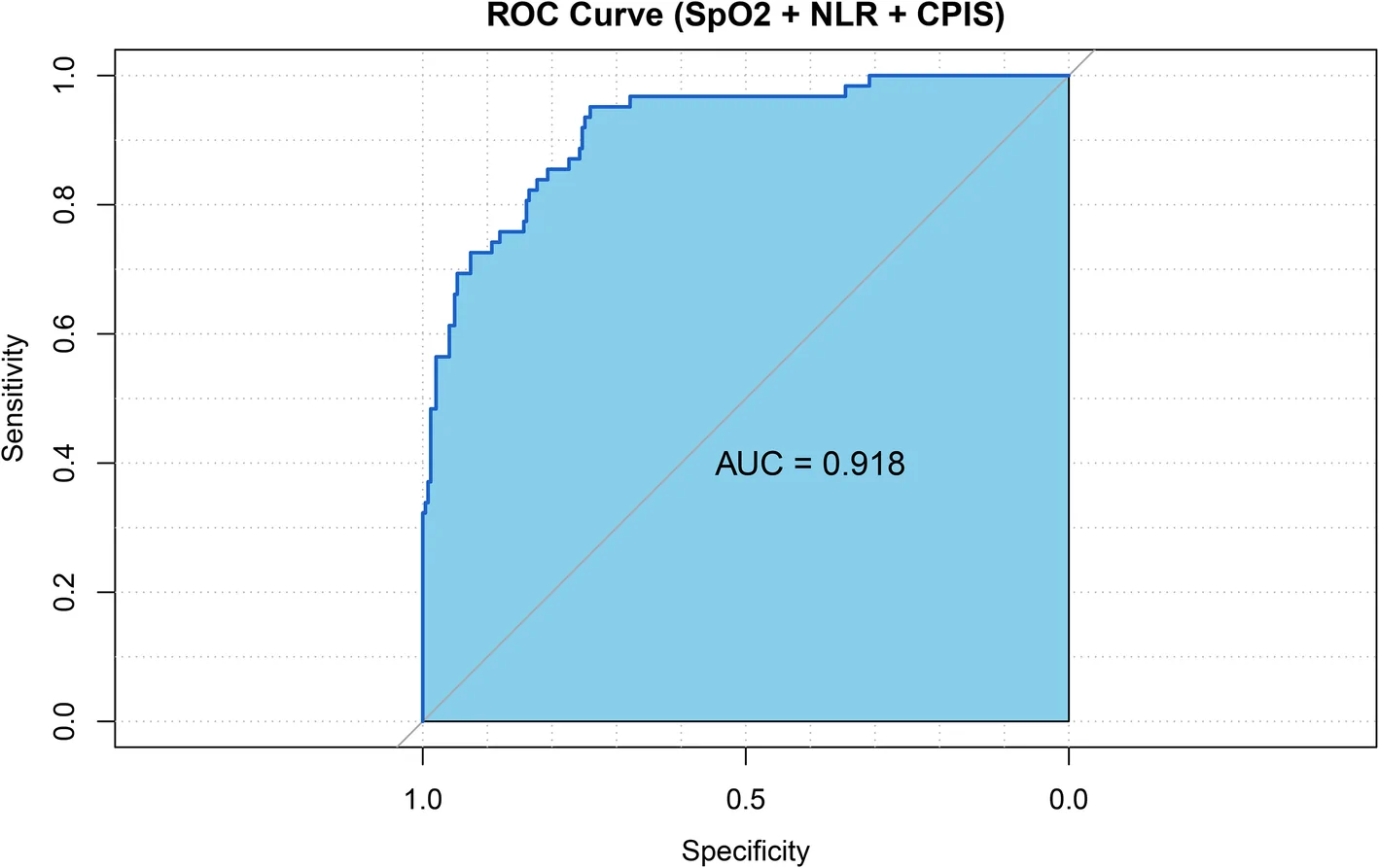
ROC curve demonstrated the clinical predictive efficacy of the respiratory failure model.

The receiver operating characteristic (ROC) curve demonstrated exceptional discriminative performance of the respiratory failure prediction model (Figure 3). The area under the curve (AUC) was 0.918 (95% CI: 0.880–0.956, *P* < 0.001), indicating excellent ability to distinguish between patients who developed respiratory failure and those who did not. At the optimal cutoff value, the model achieved a sensitivity of 92.3% and specificity of 89.5%, positive predictive value (PPV) of 68.4%, and negative predictive value (NPV) of 97.8%, further confirming its high diagnostic accuracy for clinical application.

### Model validation and clinical performance

**Figure 4:**
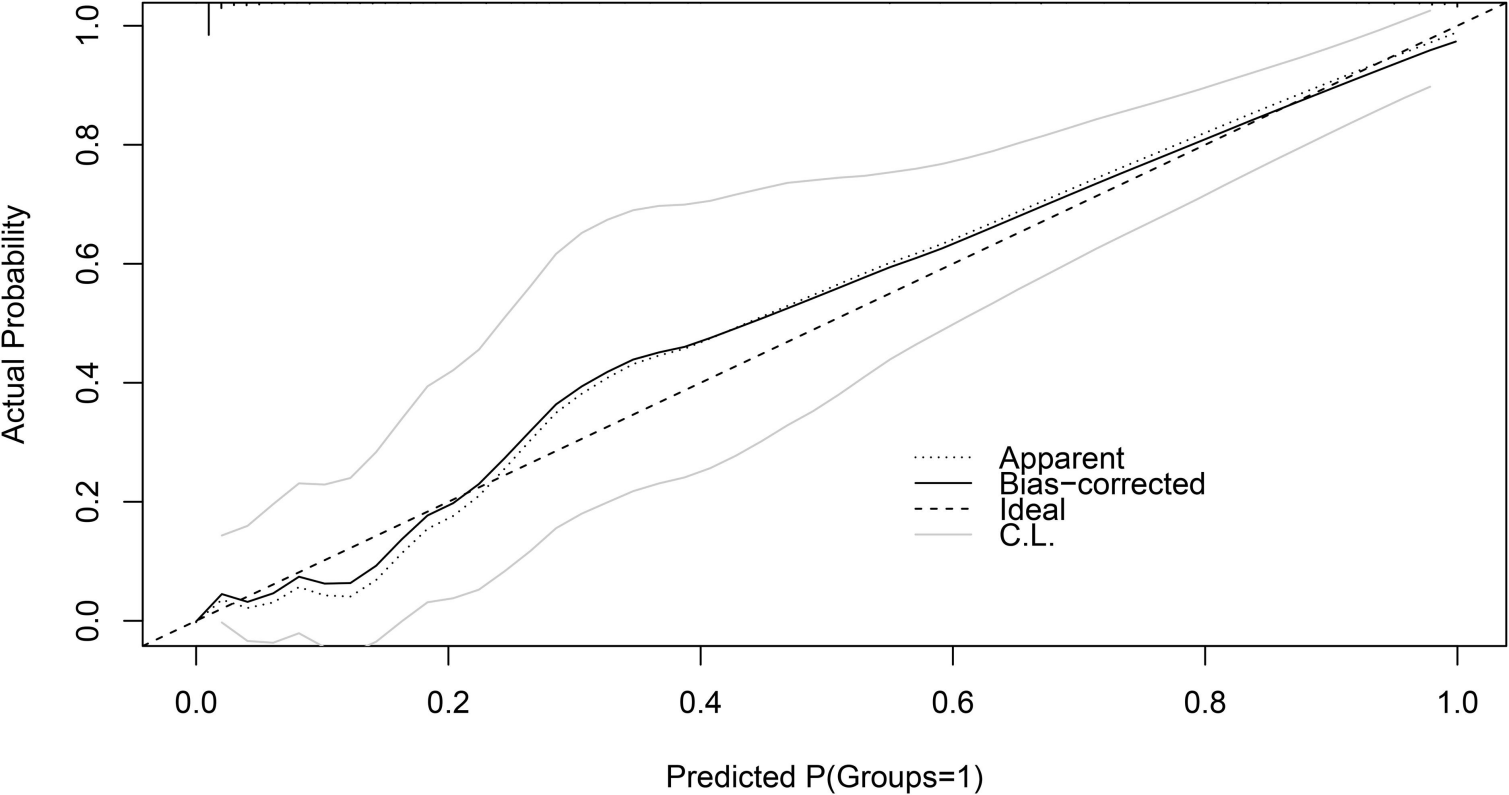
Calibration analysis of the respiratory failure model.

Calibration analysis revealed strong agreement between predicted probabilities and observed outcomes (Figure 4). Both apparent and bias-corrected calibration curves closely aligned with the ideal 45-degree reference line across the entire range of predicted probabilities. The Hosmer-Lemeshow test confirmed adequate model calibration (*χ*^2^ = 5.42, *P* = 0.71).

**Figure 5:**
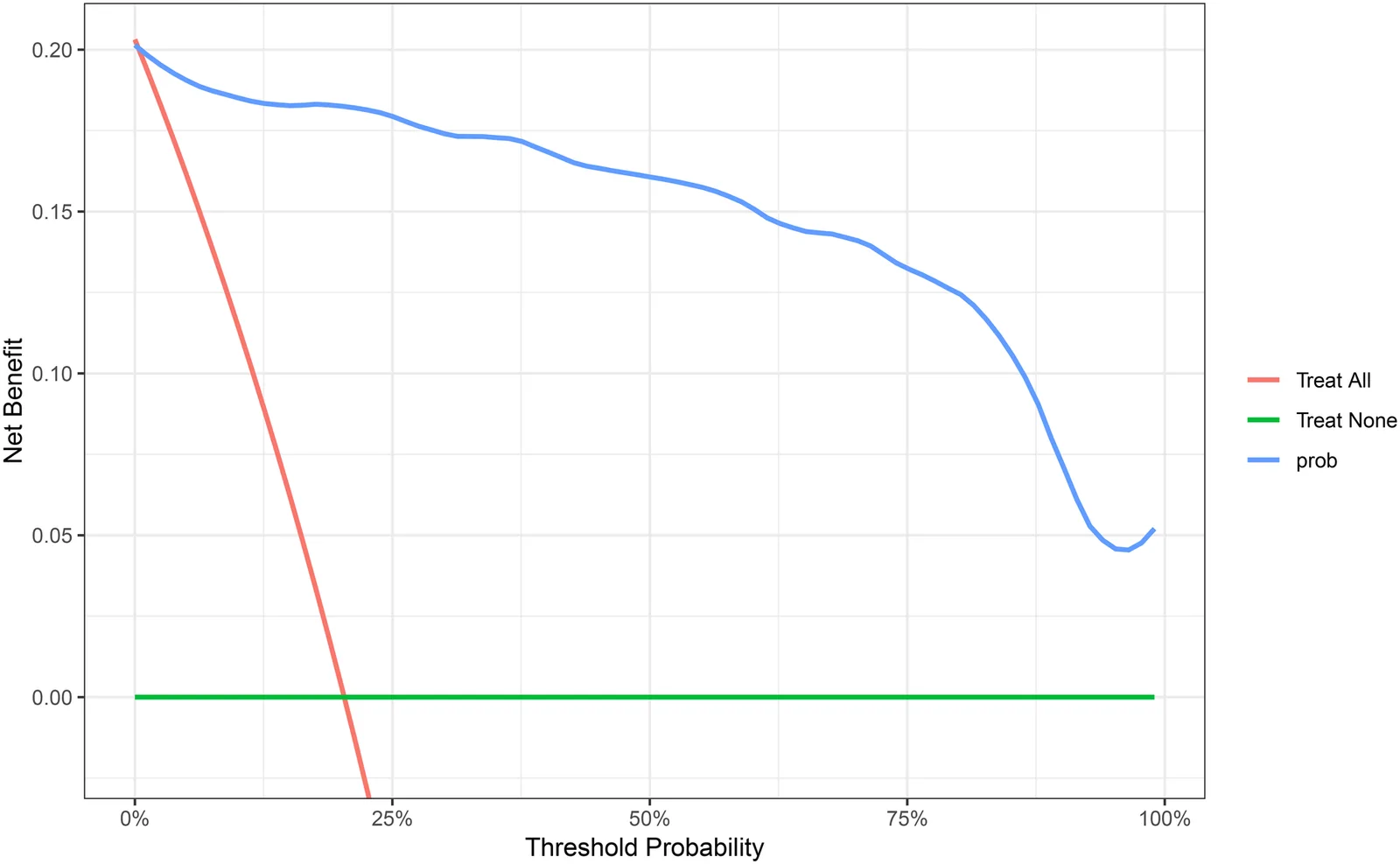
Decision curve analysis of the respiratory failure model.

Decision curve analysis demonstrated positive net benefit across a broad range of threshold probabilities (0.05–0.40) (Figure 5). At a 20% risk threshold, the model yielded a net benefit of 0.18, substantially exceeding both “classify-all” and “classify-none” strategies while maintaining superior performance up to a 35% threshold. These findings confirm the model's clinical applicability for risk stratification and early identification of high-risk patients requiring enhanced monitoring.

### Sensitivity analysis addressing predictor–outcome overlap

To evaluate the extent to which the model's discriminative performance was influenced by the conceptual overlap between oxygenation-related predictors (SpO_2_ and the PaO_2_/FiO_2_ component of CPIS) and the outcome definition (PaO_2_/FiO_2_ < 300 mmHg), we performed a sensitivity analysis using a reduced model that excluded these overlapping parameters. In this reduced model, the modified CPIS was recalculated on a 0–8 point scale (excluding the oxygenation component), and SpO_2_ was omitted entirely, leaving age, NLR, and interstitial changes as the remaining predictors. After 1,000 bootstrap resamples, the reduced model achieved an AUC of 0.860 (95% CI: 0.812–0.908). Although this represents a decline from the full model's AUC of 0.918, the reduced model still demonstrated good discriminative ability, with its AUC remaining superior to most previously reported pediatric pneumonia severity scores (typically AUC 0.70–0.80). These findings suggest that while oxygenation-related parameters do contribute to the full model's performance, the predictive signal is not solely attributable to definitional overlap, and the non-oxygenation predictors (age, NLR, and interstitial changes) carry substantial independent prognostic value.

## Discussion

This study developed and validated a robust predictive model for respiratory failure in children with Mycoplasma pneumoniae pneumonia (MPP), identifying interstitial changes on chest imaging, neutrophil-to-lymphocyte ratio (NLR), and Clinical Pulmonary Infection Score (CPIS) as independent risk factors, while demonstrating excellent discriminative performance (AUC = 0.918). The nomogram constructed from these parameters provides a clinically practical tool for early risk stratification and targeted intervention.

Our finding that interstitial changes on chest imaging independently predict respiratory failure aligns with previous studies demonstrating that interstitial lung abnormalities (ILA) identified by chest CT may represent early or mild pulmonary fibrosis and are associated with respiratory function deterioration and poor prognosis (10). A previous study reported that acute interstitial infiltration can be used for the diagnosis of pneumonia, and the degree of infiltration is related to inflammatory markers (11). This radiographic pattern likely reflects the underlying pathophysiology of pneumonia, where Mycoplasma pneumoniae triggers a hyperinflammatory cascade causing alveolar-capillary membrane injury and interstitial edema (12). In severely affected MPP patients, ultrastructural changes such as thickening of the alveolar septum, edema of the alveolar membrane, and vacuolation of alveolar epithelial cells can be observed (13). The presence of interstitial changes may indicate advanced lung injury that compromises gas exchange efficiency, predisposing patients to respiratory failure. Most current studies concentrate on adult patients and different lung conditions, but our research bridges the gap concerning pediatric MPP.

This study found that the levels of white blood cell count, NLR, CRP and D-dimer in children with respiratory failure were significantly higher than those in the non-respiratory failure group, which is consistent with the results of previous studies. The inflammatory response and activation status of immune cells in severely affected MPP patients are key indicators of disease progression, which is consistent with the elevated NLR and CRP levels observed in this study. NLR, as a sensitive indicator of systemic inflammatory response, may increase if the child's immune system has been overactivated, which in turn leads to lung tissue damage and deterioration of respiratory function (14). Furthermore, an increase in D-dimer levels indicates abnormal coagulation function and microthrombus formation, which may be one of the potential mechanisms underlying respiratory failure in MPP patients (15). However, although the platelet count was higher in the respiratory failure group in this study, it did not reach statistical significance. This might be due to the heterogeneity of platelet responses in MPP patients or the limited statistical power caused by the insufficient sample size.

This study found a significant negative correlation between CPIS and SpO_2_ (r = −0.572), indicating that the higher the CPIS score, the worse the oxygenation status of the children. This finding was consistent with the purpose of using CPIS to assess the severity of lung infections in clinical practice (16). The significant positive correlation between CPIS and NLR (*r* = 0.585) further supports the role of the inflammatory response in MPP respiratory failure. It is worth noting that CPIS is negatively correlated with D-dimer (*r* = −0.443), which may reflect the complex relationship between coagulation dysfunction and the severity of pulmonary infection. D-dimer is a product of fibrin degradation, and a significant increase in its level is a sign of active thrombosis and hyperfibrinolysis in the body (17). Thrombosis and inflammatory responses will increase pulmonary vascular permeability, leading to pulmonary edema and further impairing gas exchange. Hypoxemia is an important predictor of death in children with pneumonia (18, 19). In this study, the negative correlation between SpO_2_ and NLR suggests that the inflammatory response may exacerbate the condition by affecting the oxygenation function (16). This finding is consistent with the findings that support the hypothesis regarding the activation of immune cells and the progression of the disease (14). In MPP, CPIS may reflect the cumulative effect of alveolar infiltration and inflammatory exudates on oxygenation. Our correlation study results indicate that as CPIS increases, oxygen saturation decreases proportionally, while systemic inflammation (NLR) intensifies, forming a vicious cycle that accelerates respiratory failure.

Through single-factor and multi-factor logistic regression analysis, this study identified the independent risk factors for respiratory failure in children with MPP, and constructed a nomogram prediction model including interstitial changes on imaging, NLR, CPIS, age and SpO_2_. CPIS integrates imaging, oxygenation and microbiological data, and may be more applicable to diseases like MPP where the imaging manifestations are diverse. However, the feasibility of complex scoring tools in primary healthcare is limited, so this model simplifies the application of CPIS through a nomogram visualization, aligning with the “simplified risk stratification” goal (20). Younger age and higher SpO_2_ levels are associated with a reduced risk of respiratory failure. Previous studies indicated that children aged 6–14 years with MPP had lower rates of severe disease compared to preschool-age children (2, 21). The protective effect of SpO_2_ is consistent with previous studies. Hypoxemia is a strong predictor of mortality risk, and active intervention is required when SpO_2_ is less than 93% (22). It is worth noting that in adult MPP studies, it was found that glucocorticoids have an improving effect on hypoxemia. However, there is limited data for children. This model provides support for the importance of oxygenation monitoring in the pediatric population. Compared with multi-national CAP prediction models (23), this study focuses on MPP-specific indicators (such as interstitial changes, NLR), which compensate for the previous models' insufficient consideration of pathogen heterogeneity and incorporate more routine indicators that better meet the needs of grassroots settings.

This study has several limitations that warrant consideration. First, the single-center retrospective design introduces the possibility of selection bias and limits the generalizability of the findings to other populations and healthcare settings. MPP epidemiology, circulating genotypes, macrolide resistance rates, and disease severity vary substantially across regions and epidemic seasons, so the model's discrimination and calibration may not directly translate to other settings or time periods. External validation in independent multicenter prospective cohorts is essential before widespread clinical implementation. Second, and most critically, we acknowledge a methodological concern regarding conceptual overlap between certain predictors and the outcome definition. The respiratory failure outcome included a PaO_2_/FiO_2_ < 300 mmHg criterion, while the modified CPIS inherently contains an oxygenation subscore, and SpO_2_ is itself a direct marker of oxygenation status. This overlap may have partially inflated the full model's apparent AUC of 0.918. Our sensitivity analysis yielded a more conservative AUC of 0.860. This finding indicates that the true predictive performance of the model likely lies between these two estimates. Nevertheless, the reduced model retained robust discriminative ability, confirming that non-oxygenation predictors—age, NLR, and interstitial changes—carry substantial independent prognostic value. We retained SpO_2_ and the CPIS in the primary model because SpO_2_ is a universally available, non-invasive, real-time parameter that clinicians routinely use in triage decisions, and excluding it would sacrifice ease of use for statistical purity—a trade-off that may limit adoption in resource-limited settings. However, we emphasize that the model AUC of 0.918 should be interpreted with caution, and external validation is urgently needed to obtain unbiased estimates of the model's true discrimination in routine practice. Third, the exclusion of patients with concurrent bacterial or viral co-infections, while methodologically necessary to isolate the effects of MPP, substantially limits the model's generalizability to real-world clinical settings where mixed infections are common, particularly during outbreak seasons. Therefore, our model is validated only for “uncomplicated MPP”; its performance in children with MPP and coinfections remains entirely unknown and should be a priority for future investigation. Fourth, the relatively small number of respiratory failure events (62 cases) may increase the risk of model overfitting, despite our use of 1,000 bootstrap resamples for internal validation. The stability of the regression coefficients and the model's performance should be confirmed in larger independent cohorts. Fifth, we did not incorporate several potentially important confounders, including prior antibiotic use, comorbidities, treatment timing before presentation, or pathogen genotypes and macrolide resistance status, all of which may influence disease progression and outcome. Future studies combining multi-center prospective designs with multi-omics analyses are warranted to further validate and extend our findings. During the MPP outbreak, the rate of co-infection was high, but this model did not assess the impact of co-infecting pathogens. Additionally, the data on pathogen genotypes or drug resistance were not included, which limited the generalizability of the results. In the future, a multi-center prospective study combined with multi-omics analysis can be conducted to further verify and expand the conclusions of this study.

## Conclusion

This study, based on the MPP respiratory failure prediction model established by CPIS, integrates imaging, inflammatory and clinical indicators, providing a basis for the risk stratification of respiratory failure in MPP children. The nomogram translates these findings into a practical bedside tool that enables clinicians to identify high-risk children early, allocate intensive care resources efficiently, and potentially improve outcomes through preemptive respiratory support.

## Data Availability

The raw data supporting the conclusions of this article will be made available by the authors, without undue reservation.
